# A Case of Severe Aplastic Anemia in a 35-Year-Old Male With a Good Response to Immunosuppressive Therapy

**DOI:** 10.7759/cureus.40210

**Published:** 2023-06-10

**Authors:** Ekaterina Proskuriakova, Ranjit B Jasaraj, Aleyda M San Hernandez, Anuradha Sakhuja, Mtanis Khoury, Pam Khosla

**Affiliations:** 1 Internal Medicine, Mount Sinai Hospital, Chicago, USA; 2 Hematology and Oncology, Mount Sinai Hospital, Chicago, USA

**Keywords:** eltrombopag, anti-thymocyte globulin (atg), hypocellular bone marrow, paroxysmal nocturnal hemoglobinuria (pnh), severe aplastic anemia

## Abstract

Aplastic anemia (AA) is a severe but rare hematologic condition associated with hematopoietic failure leading to decreased or total absent hematopoietic precursor cells in the bone marrow. AA presents at any age with equal distribution among gender and race. There are three known mechanisms of AA: direct injuries, immune-mediated disease, and bone marrow failure. The most common etiology of AA is considered to be idiopathic. Patients usually present with non-specific findings, such as easy fatigability, dyspnea on exertion, pallor, and mucosal bleeding. The primary treatment of AA is to remove the offending agent. In patients in whom the reversible cause was not found, patient management depends on age, disease severity, and donor availability. Here, we present a case of a 35-year-old male who presented to the emergency room with profuse bleeding after a deep dental cleaning. He was found to have pancytopenia on his laboratory panel and had an excellent response to immunosuppressive therapy.

## Introduction

Aplastic anemia (AA) is a rare condition characterized by the combination of hypoplasia or aplasia of the bone marrow and pancytopenia in at least two of the three main lines of cells: red blood cells (RBCs), white blood cells (WBCs), and platelets [1]. An estimated incidence of this disease is 0.6 to 6.1/million per year with a sex ratio of about 1:1 [2]. AA is more common in Asia than in Western countries [3]. This could reflect the variability of exposure to different environmental factors, such as drugs, chemicals, viral pathogens, or genetic predisposition. Although this condition could be seen in any age group, two incidence peaks of AA are reported: in young adults (20-25 years old) and the elderly population with a peak after the age of 60 [4,5].

The three main mechanisms of AA are direct injuries, immune-mediated disease, and bone marrow failure (inherited or acquires) [1]. The most common etiology of AA is considered to be idiopathic, responsible for 65% of cases. Seronegative hepatitis accounts for about 10% of cases and tends to develop three months after the episode of acute hepatitis [6]. Telomerase abnormalities are found in approximately 5% of late-onset AA [7]. Among hereditary causes, Fanconi anemia is the most common, which presents in the first 10 years of life with pancytopenia, hypoplasia, and bone abnormalities [8].

The clinical manifestation of AA is usually some non-specific finding due to pancytopenia, such as fatigue, dyspnea on exertion due to anemia, mucosal bleeding like petechiae, heavy menses, gingival bleeding due to thrombocytopenia, or fever with neutropenia [9]. A bone marrow examination with a finding of aplastic or hypoplastic marrow is required to establish a diagnosis. In addition, cytogenetic studies, such as fluorescence in situ hybridization (FISH) or next-generation sequencing (NGS), help make a diagnosis and rule out other hematologic abnormalities responsible for pancytopenia [9]. Peripheral blood-flow cytometry could be helpful in order to exclude paroxysmal nocturnal hemoglobinuria (PNH) [10].

Management of AA in patients without reversible causes depends on the age of the patient and disease severity. For young and healthy individuals under 50 years old, an allogeneic hematopoietic cell transplant (HCT) should be performed before initiation of immunosuppressive therapy (IST). Those who are older than 50 years old or younger individuals who cannot have HCT should start on full-dose IST, including eltrombopag, which is a thrombopoietin agonist, anti-thymocyte globulin (ATG) that eliminates antigen-reactive T-cells, cyclosporin A inhibiting interleukin-II (IL-2), and prednisone that leads to the destruction of immature T-lymphocytes [9]. Supportive treatment with transfusions of leukoreduced RBC for hemoglobin (Hgb) less than 7 mg/dL or platelets less than 10,000/microliters and infection treatment or prophylaxis is also indicated for patients with AA [1].

Here, we present a case of a 35-year-old male who presented to the emergency room with profuse bleeding after deep dental cleaning and was found to have pancytopenia on his laboratory panel.

## Case presentation

A 35-year-old male presented to the emergency department (ED) of our hospital for persistent bleeding of his gums. He had been having episodes of minimal gum bleeding for a week that he attributed to an infection and had gone to a dentist on the day of admission for dental cleaning. He started having profuse bleeding after the dental procedure, which did not resolve with the application of pressure and was advised to go to the ED.

At the time of admission, he was in mild distress and concerned about the bleeding. The patient did not have any dizziness, headache, palpitations, or bleeding anywhere else. He denied similar episodes in the past or a family history of bleeding. The patient denied tobacco, alcohol, or illicit drug use and was not on any anticoagulants/anti-platelets. The patient migrated from Mexico 20 years ago and worked in construction. He denied any sick contact or recent travel. On examination, his vital signs were stable, but he was in mild distress. He was having profuse bleeding in his bilateral lower gums, both the buccal and lingual side, and in the buccal side of his upper gums. The rest of the examination was unremarkable.

Initial laboratory results were significant for pancytopenia (Table 1). The chemistry panel was significant for elevated blood urea nitrogen and mild hypokalemia (potassium = 3.4 mEq/L). The liver function test, renal function test, coagulation panel, and other electrolyte results were normal. The peripheral blood smear test showed normal WBC and platelet morphology, decreased platelet number, abnormal RBC morphology, marked hypochromasia, and slight schistocytes.

**Table 1:** CBC results. g/dL: grams per deciliter; mm^3^: cubic meter; µm^3^: cubic micrometer; pg/cell: picogram per cell CBC: complete blood count; WBCs: white blood cells; RBCs: red blood cells; Hbg: hemoglobin; Hct: hematocrit; MCV: mean cell volume; MCH: mean cell hemoglobin; PT: prothrombin time; INR: international normalized ratio; PTT: partial thromboplastin time

	On admission	Range
WBCs	2.7/mm^3^	(4-11)/mm^3^
RBCs	2.07 million/mm^3^	(4.34-5.6) million/mm^3^
Hbg	7 g/dL	(13.5-17.5) g/dL
Hct	19.2%	(38.6-49.2)%
MCV	93 µm^3^	(80-11) µm^3^
MCH	34.1 pg/cell	(26-34) pg/cell
Platelets	4/mm^3^	(150-450)/mm^3^
PT	13.7 seconds	(11.9-15.0) seconds
INR	1	
PTT	20.5 seconds	(24.8) seconds

Within two hours, the patient became hypotensive and developed hemorrhagic shock, and was admitted to the intensive care unit. The patient’s mouth was packed, and he received multiple transfusions of packed RBC and platelets. Further test results (Table 2) were negative for any infections. However, he was positive for parvovirus and cytomegalovirus IgG antibodies, which were likely from a previously cleared infection. The reticulocyte count was 0.9 after correction for hematocrit, and haptoglobin and lactate dehydrogenase (LDH) were both within normal limits, pointing toward the hypoproliferation of the bone marrow rather than hemolysis.

**Table 2:** Viral serology profiles. HIV: human immunodeficiency virus; HBVs Ag: hepatitis B surface antigen; HBVc IgM Ab: IgM antibody against hepatitis B core antigen; HBV cIgM: hepatitis B virus cytoplasmic IgM; HCV IgG Ab: IgG antibody against hepatitis C; HAV IgM: IgM antibody against hepatitis A; COVID-19: coronavirus disease 2019; CMV IgG Ab; IgG antibody against cytomegalovirus; CMV IgM Ab: IgM antibody against cytomegalovirus; parvovirus B19 IgG Ab: parvovirus B19 IgG antibody; parvovirus B19 IgM Ab: parvovirus B19 IgM antibody

HIV	Negative
HBVs Ag	Non-reactive
HBVc IgM Ab	Non-reactive
HBV cIgM	Non-reactive
HCV IgG Ab	Non-reactive
HAV IgM	Non-reactive
COVID-19	Negative
CMV IgG Ab	>8 H
CMV IgM Ab	<0.2
Parvovirus B19 IgG Ab	1.95 H
Parvovirus B19 IgM Ab	<0.34

**Figure 1:**
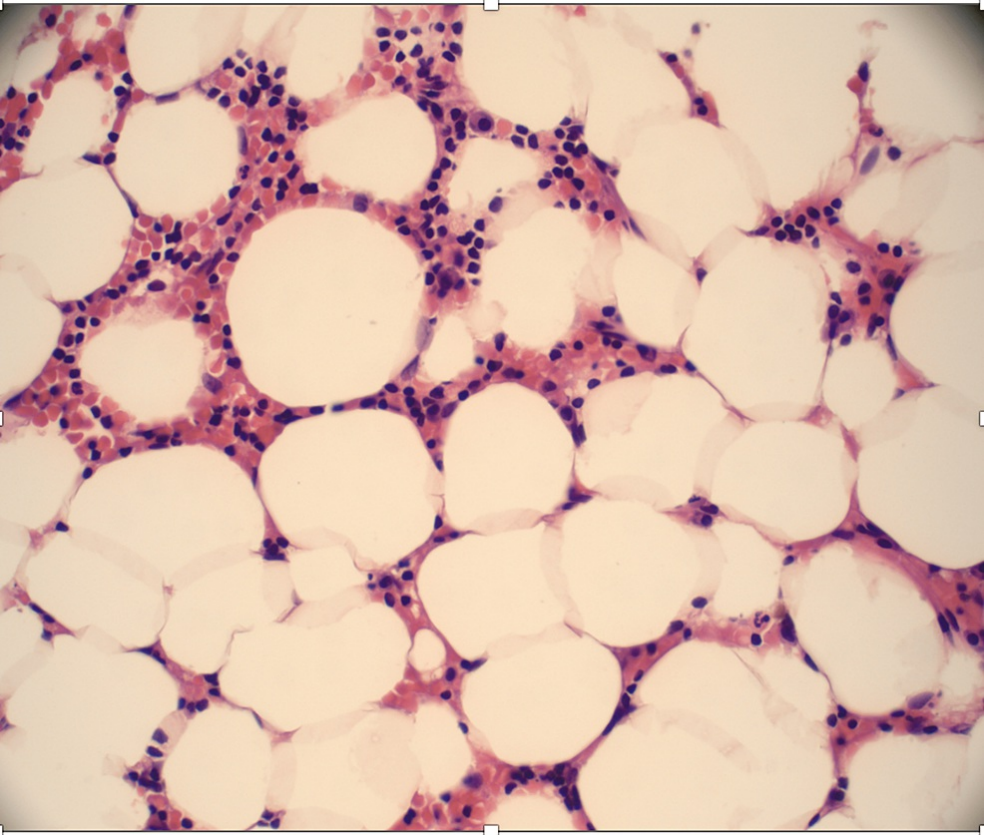
Bone marrow aspiration is aparticulate and markedly hypocellular, 5-10% cellularity consistent with aplastic anemia. No abnormal cells were seen.

**Figure 2:**
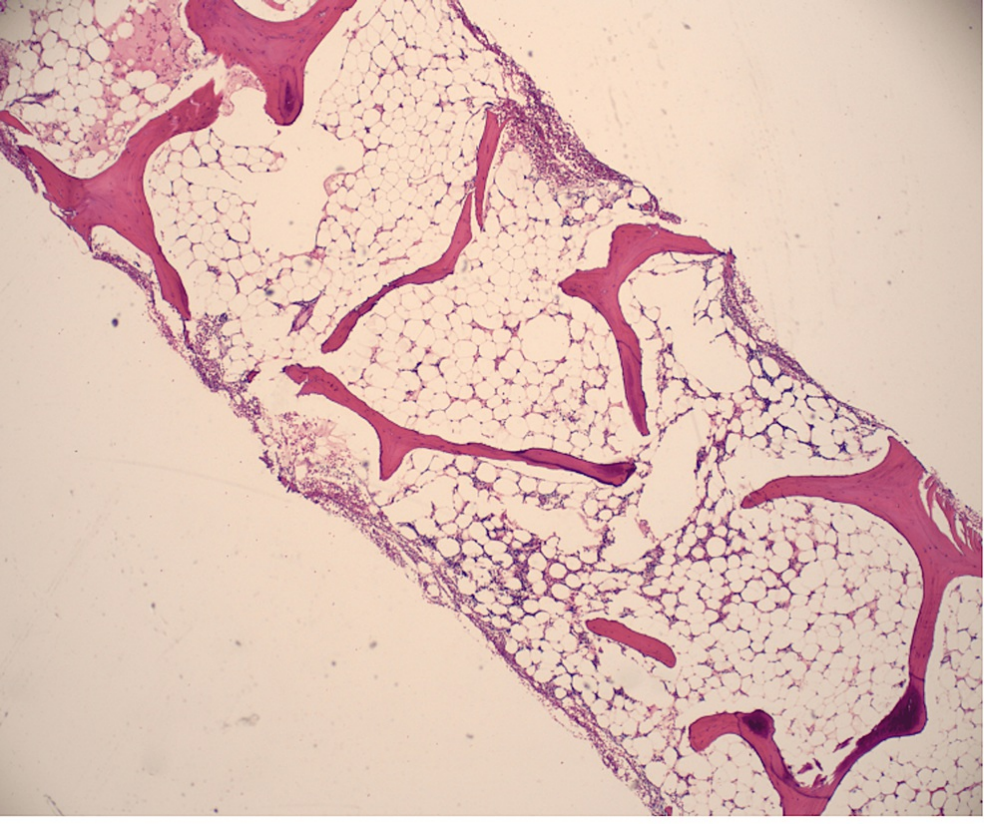
Bone marrow trephine biopsy shows a hypocellular marrow, which was replaced mainly with nonhematopoietic tissues, such as fat.

The bone marrow biopsy showed a hypocellular bone marrow with 5-10% cellularity consistent with AA (Figure 1, Figure 2).

PNH flow cytometry with fluorescein-labeled proaerolysin (FLAER), which is a high-sensitivity assay that assesses the glycosylphosphatidylinositol (GPI)-linked CD59 on erythrocytes, was abnormal with elevated PNH monocytes (2.001%) and polymorphonuclear neutrophil (PMNs) (0.381%). No circulating blasts and megakaryocytes were seen. The overall AA is associated with PNH.

The patient was not accepted for the Allogenic Hematopoietic Stem Cell Transplant (HSTC) Center due to the lack of insurance. The patient was started on triple immune suppression therapy: ATG, cyclosporine (CsA), and prednisone. The patient was started on equine ATG 40 mg/kg/day IV for four consecutive days in combination with CsA 10 mg/kg every 12 hours and prednisolone (0.5 mg/kg/day). In addition, he received eltrombopag 150 mg per oral (PO) once per day, an oral thrombopoietin-receptor agonist, and diphenhydramine 25 mg to prevent serum sickness from ATG. The patient continued to be on neutropenic precautions and close monitoring of all cell line levels, with goals of Hgb 7 g/dL and platelet count 10,000/mm^3^.

The patient was discharged home to continue outpatient chemotherapy. On the last follow-up at the outpatient clinic, three months after admission, recovery of all the three cell lines was noted (Table 3).

**Table 3:** CBC results. CBC: complete blood count; WBCs; white blood cells; RBCs: red blood cells; Hbg: hemoglobin; Hct: hematocrit; MCV: mean cell volume; MCH: mean cell hemoglobin

	On admission	Three months later	Normal range
WBCs	2.7/mm^3^	4.2/mm^3^	(4-11)/mm^3^
RBCs	2.07 million/mm^3^	2.65 million/mm^3^	(4.34-5.6) million/mm^3^
Hbg	7 g/dL	8.1 g/dL	(13.5-17.5) g/dL
Hct	19.2%	30.4%	(38.6-49.2)%
MCV	93 µm^3^	86 µm^3^	(80-11) µm^3^
MCH	34.1 pg/cell	35.4 pg/cell	(26-34) pg/cell
Platelets	4/mm^3^	18/mm^3^	(150-450)/mm^3^

## Discussion

The 35-year-old patient with no significant past medical history presented to the hospital with oropharyngeal bleeding. The severity of his thrombocytopenia expanded and the bleeding worsened upon his presentation to the hospital. His decreased WBC count and neutropenia put him at a high risk of infection. Low Hgb and below-normal reticulocytes showed that his RBC production was profoundly impaired. The bone marrow biopsy ruled out other differential diagnoses, but the cause of AA development was still questionable. In addition, the patient was found to have PNH, which could be associated with AA in 40% of patients. However, there are contradictory data in the literature about the impact of PNH clones on patients with AA undergoing immunosuppressive treatment.

AA can be associated with a broad spectrum of pathologies that could lead to the loss of progenitor cells and pancytopenia. There are three main mechanisms of its development: disrupting extrinsic factors, expression of familial genetic mutations, or damage by an autoimmune attack on hematologic cells [1]. The extrinsic mechanisms of AA are usually apparent and include exposure to therapeutic radiation, benzene, chemotherapy [11], several medications, or pesticides, such as organophosphates [7]. Nevertheless, this patient did not take any medications or have no known history of radiation or chemotherapy.

A genetic abnormality that is mainly associated with AA is Fanconi anemia, a condition attributed to DNA repair abnormalities. This syndrome is usually manifested in patients during the first or second decade with other congenital defects, such as thumb or facial abnormalities and short stature [12]. Dyskeratosis congenita is another common mechanism, a condition caused by mutations in genes responsible for the repair of telomeres. Patients can present with skin pigmentations, oral leukoplakia, and dystrophic nails [13]. Congenital abnormality was highly unlikely in this patient; he did not have any family history of such conditions, nor had a clinical manifestation.

Another common cause of AA can be seronegative hepatitis, which can develop in up to 10% of cases approximately three months before the manifestation of AA [14]. It usually occurs in the younger population. However, this patient did not have any risk factors or any history related to the development of hepatitis. Other viruses that could predispose to AA include HIV or parvovirus B19 [15]. IgG usually develops two weeks after infection and persists for life, with an increase in level post-re-exposure. Transient aplastic crisis (TAC) is characterized by the abrupt onset of anemia with absent or low-level reticulocytes and can be associated with B19 in patients with hematologic abnormalities. TAC and B19 can also lead to other cytopenias in other blood lineages [16]. TAC could be a potential explanation for the patient’s symptoms as his blood tests revealed elevated IgG levels to B19. This finding can reflect only previous infections and has no association with AA in this patient. Nevertheless, regardless of the trigger, the patient’s presentation and the severity of his AA were associated with a high chance of death without urgent management.

Management of AA depends on the severity of the condition, the age of a person, access to the treatment or availability of a matched stem-cell donor, and the presence of other comorbidities that decrease the chance of getting a cell transplant [17]. The patient’s laboratory values met the criteria for severe AA, which are defined by bone marrow hypocellularity of less than 30% and involvement of at least two out of the three criteria: absolute reticulocyte count less than <60 ×109/L, absolute neutrophil count less than 0.5×109/L, or platelet count less than 20 ×109/L. Younger age (less than 40) and the presence of a matched sibling donor favor the use of the allogeneic HSCT. Usually, older patients (>40 years) and those who do not have access to HSCT are treated with IST, which includes a combination of ATG and CsA with response rates up to 80% and survival rates the same as those after HSCT [18]. However, rates of relapse and evolution to myelodysplastic syndromes are higher with IST treatment [19]. Bacigalupo et al. showed an overall survival rate of 87% and a response rate of 77% in 100 patients treated with CsA, ATG, prednisone, and filgrastim [20]. Nevertheless, the patient did not have access to the treatment with an allogeneic stem cell transplant. He was commenced on full IST with eltrombopag, ATG, and CsA with prednisone and diphenhydramine to prevent serum sickness from ATG.

Some studies have revealed factors that predict a better response to IST, such as younger age, high absolute reticulocyte and lymphocyte count, and mutations in the PIGA, BCOR, or BCORL1 genes [21]. Mutations in the PIGA gene lead to the lack of the GPI-anchored proteins, CD59, and decay-accelerating factor (DAF or CD55) in patients with PNH. Deficiency of these GPI-anchoring proteins in WBCs and RBCs leads to the development of the so-called escape clones of PNH in AA [22].

The explanation of this immune escape mechanism is proposed to be associated with cytotoxic T cells that target normal hematopoietic stem cells (HSCs), and PNH-positive HSCs are protected from this immune attack. These T cells’ target can most likely be GPI anchors in patients with AA [23,24].

PNH escape clones can be found in more than 50% of patients with AA at the time of diagnosis, as in this patient. According to the AA guidelines of the British Society for Standards in Haematology, all patients with AA should be screened for PNH clones [17]. The impact of PNH clones on the treatment outcome of patients treated with IST has been discussed in the recent meta-analysis that showed a better response rate in the group diagnosed with PNH [25].

Apart from IST, patients with AA usually require supportive care that includes prophylaxis and treatment of infections, transfusions of leukoreduced packed RBC if the level of Hgb is less than 7 mg/dL, or platelets if their level drops below 10 x 109/L, or less than 50 x 109/L with active bleeding [1]. The patient presented with a platelet count of 4 x 109/L; therefore, he received multiple platelet transfusions before being discharged home.

The patient gradually improved on IST and was eventually discharged home to continue outpatient chemotherapy. On the last follow-up at the outpatient clinic, three months after admission, recovery of all the three cell lines was noted.

## Conclusions

In this case report, we described a case of a healthy young man with no medical history who presented to the hospital with persistent bleeding from his gums and was found to have AA. The patient’s laboratory values met the criteria for severe AA, and he was also found to have PNH that could be associated with AA in 40% of patients. Patients with age less than 40 and the presence of a matched sibling donor favor the use of HSCT. Our patient did not have access to the bone marrow transplant. Therefore, he was commenced on full IST with ATG, prednisone, and CsA. In addition to IST, the patient was also receiving eltrombopag. The patient with severe AA responded excellently to the therapy, recovering all the three cell lines after three months of management. Therefore, young age, no significant past medical history, and concomitant diagnosis with PNH can be the main factors leading to a better response rate to IST in patients diagnosed with severe AA.
